# Characterization of presumptive vancomycin-resistant enterococci recovered during infection control surveillance in Dallas, Texas, USA

**DOI:** 10.1099/acmi.0.000214

**Published:** 2021-03-22

**Authors:** Sara Ping, Nancy Mayorga-Reyes, Valerie J. Price, Michelle Onuoha, Pooja Bhardwaj, Marinelle Rodrigues, Jordan Owen, Dennise Palacios Araya, Ronda L. Akins, Kelli L. Palmer

**Affiliations:** ^1^​ Department of Biological Sciences, The University of Texas at Dallas, Richardson, Texas, USA; ^2^​ Methodist Charlton Medical Center, Dallas, Texas, USA

**Keywords:** *Enterococcus*, molecular typing, vancomycin-resistant enterococci

## Abstract

*
Enterococcus faecalis* and *E. faecium* are Gram-positive bacteria that normally inhabit the human gastrointestinal tract. They are also opportunistic pathogens and can cause nosocomial infection outbreaks. To prevent the spread of nosocomial infections, hospitals may rely on screening methods to identify patients colonized with multidrug-resistant organisms including vancomycin-resistant enterococci (VRE). Spectra VRE agar (Remel) contains vancomycin and other medium components that select for VRE and phenotypically differentiate between *E. faecalis* and *E. faecium* by colony colour. We obtained 66 de-identified rectal swab cultures on Spectra VRE agar that were obtained during routine patient admission surveillance at a hospital system in Dallas, Texas, USA. We analysed 90 presumptive VRE from 61 of the Spectra VRE agar cultures using molecular and culture methods. Using *ddl* typing, 55 were found to be *E. faecium* and 32 were found to be *E. faecalis *. While most of the *E. faecium* were positive for the *vanA* gene by PCR (52 of 55 strains), few of the *E. faecalis* were positive for either *vanA* or *vanB* (five of 32 strains). The 27 *E. faecalis vanA*- and *vanB*-negative strains could not be recultured on Spectra VRE agar. Overall, we found that Spectra VRE agar performed robustly for the identification of vancomycin-resistant *E. faecium *, but presumptive false positives were obtained for vancomycin-resistant *E. faecalis *.

## Introduction

Vancomycin-resistant enterococci (VRE), typically *Enterococcus faecium* and less commonly *Enterococcus faecalis *, are hospital-associated pathogens of significant public health concern. VRE are among the antibiotic-resistant pathogens identified by the United States Centers for Disease Control and Prevention (CDC) as being serious threats to public health [1].

It is important to distinguish VRE from vancomycin-susceptible enterococci (VSE). VSE are normal colonizers of the human gastrointestinal tract [2]. Like VRE, VSE can opportunistically cause infections, but more treatment options are available for infections with VSE. There are a number of surveillance methods that can be employed to identify patients colonized with VRE and to guide infection control practices in clinical settings. These methods typically involve culture-based screening of rectal swabs or faecal material in media containing vancomycin. One such medium is Spectra VRE agar (Remel). Spectra VRE agar contains vancomycin at 6 µg ml^−1^ and proprietary chromogens that allow distinction between *E. faecium* and *E. faecalis* based on colony colour of navy blue or purple and light blue, respectively.

In this study, we analysed presumptive VRE obtained using Spectra VRE medium during routine patient admission screening for multidrug-resistant organisms (MDROs) at a hospital system in Dallas, Texas, USA. Our short-term goal specific for this study was to validate the isolates obtained from Spectra VRE agar cultures using molecular typing techniques. Our long-term goal, outside the scope of this study, is to use genome sequencing approaches to study the phylogeny and antibiotic resistance of these isolates.

## Methods

### Culture methods and molecular biology procedures

Unless otherwise stated, enterococci were cultured at 37 °C in brain heart infusion (BHI) broth or on BHI agar. Spectra VRE agar plates for independent testing of presumptive VRE isolates were purchased from Fisher Scientific. Genomic DNA was isolated using the DNeasy Blood and Tissue kit (Qiagen). Taq polymerase (New England Biolabs) was used for PCR. PCR products used for sequencing analysis were purified using the GeneJET PCR Purification kit (Thermo Fisher Scientific). Sequencing of PCR products was performed at the Massachusetts General Hospital CCBI Core facility.

### Collection of presumptive VRE

Presumptive VRE were collected at the Methodist Dallas Health System during a routine MDRO surveillance programme previously established and performed on all patients with the following risk factors regardless of inclusion in this study. The authors had no role in designing the surveillance strategy, including the culture medium used for surveillance. Surveillance culture screening was performed on patients admitted with at least one of these risk factors: hospitalization for two or more consecutive nights in the preceding 30 days, transferred from another medical facility, residence in a nursing home or extended/long-term care facility, or the presence of decubitus ulcer or a draining wound. We obtained rectal swab cultures that were performed as a part of these routine admission surveillance procedures. The rectal swabs were plated on Spectra VRE agar. Instead of disposal after clinical surveillance was complete, Spectra VRE plates with visible growth were coded numerically, de-identified and transferred to the University of Texas at Dallas for further analysis of presumptive VRE. One plate corresponded to one unique patient. Spectra VRE plates were collected from August 2015 to October 2015. At least one colony from each Spectra VRE plate was sub-cultured into BHI broth, incubated overnight and stored at −80 °C with 25 % glycerol. If multiple colony phenotypes were observed from one Spectra VRE plate (e.g. multiple different colony colours or morphologies), representative colonies of each phenotype were picked.

### Species determination and *van* typing

The primers used in this study are detailed in Table S1 (available in the online version of this article). *ddl* primer sets used to identify *E. faecalis* and *E. faecium *, and the type of vancomycin resistance (*vanA* or *vanB*), were as previously reported [3, 4]. In the event that an isolate was neither *E. faecium* nor *E. faecalis* by *ddl* PCR, the 16S rRNA gene was amplified using universal primers 8S and 1492R [5] and sequenced. At least 1300 non-ambiguous bases were used as query against the NCBI nr/nt database by megablast. Hits with 100 % query coverage and the highest percentage sequence identity are reported. Boiled colonies were used as a template for PCR where possible; for some isolates, purified genomic DNA was used as a template.

### Vancomycin MIC

Vancomycin susceptibility was assessed using broth microdilution. Two-fold serial dilutions of the drug were made with BHI broth in a 96-well microtitre plate. An overnight culture was diluted to an OD_600_ of 0.001, and 5 µl was used to inoculate the wells of the plate. The MIC was recorded after 24 h of incubation at 37 °C. Broth microdilution experiments were performed twice independently for each strain.

### Spectra VRE growth test

Selected stocked isolates of interest were inoculated onto Spectra VRE agar to confirm whether growth occurred. Overnight cultures in BHI broth were diluted to an OD_600_ of 0.005 in 1–3 ml fresh BHI broth, then incubated at 37 °C for 3.5 h. Then, 100 µl culture was spread plated onto Spectra VRE agar; for some isolates, 50 µl culture was spread over one half of the plate. A subset of strains were also inoculated on BHI agar supplemented with vancomycin at 6 µg ml^−1^. Plates were incubated for 24 h at 37 °C. *E. faecium* 1,231,410, a VanA-type VRE [6] and *E. faecalis* V583, a VanB-Type VRE [7, 8], were included as positive controls, yielding purple and light blue lawns, respectively.

### MLST and CRISPR-Cas analysis of selected *E. faecalis* strains

The MLST database (https://pubmlst.org/efaecalis/) and sequencing of internal fragments of seven housekeeping genes were used as previously described [9] to determine the sequence type (ST) of 11 *E. faecalis* isolates. Novel STs were submitted to the database. PCR was used to screen 23 *E. faecalis* isolates for the presence of three previously identified *E. faecalis* CRISPR loci (CRISPR1-Cas, CRISPR2 and CRISPR3-Cas) [10, 11]. Specifically, previously reported primer sets were used to screen for the presence of CRISPR1 *cas9* and CRISPR3 *cas9* [10]. Strains with negative *cas9* results were then confirmed to lack the entire CRISPR-Cas loci by using previously reported primer sets that anneal outside the conserved chromosomal locations where the loci occur [10]. CRISPR2 arrays were amplified and sequenced as previously described [10, 12]. CRISPR2 spacer sequences were compared with a previously reported CRISPR2 spacer dictionary generated from 228 *E. faecalis* genomes [12].

### Accession numbers

The 16S rRNA gene sequences for isolates 133-2 and 146 have been deposited in GenBank under accession numbers MW113739 and MW113740, respectively.

## Results

### Initial analysis of Spectra VRE cultures

We obtained 66 Spectra VRE plates with visible bacterial growth, corresponding to 66 presumptive VRE-positive patients. Subsequently, 100 colonies of interest were identified and stocked for further analysis (Table S2). Based on colony colour, 33 were presumptive *E. faecalis* VRE (light blue colour), 64 were presumptive *E. faecium* VRE (navy blue or purple) and three colonies had atypical colour (white) (Table S2).

### Confirmation of species prediction

Of the 100 stocked strains, 10 could not be revived from freezer stock (Table S2). Of these, one was a presumptive *E. faecalis* by colony colour and nine were presumptive *E. faecium* by colony colour. The inability to revive these isolates reduced our isolate number to 90 and our patient cohort size from 66 to 61.

Primer sets designed to amplify the *ddl* genes of *E. faecium* and *E. faecalis* [3, 4] were used to determine the species of the 90 presumptive VRE isolates. For strains where *ddl* PCR yielded negative results for both *E. faecalis* and *E. faecium* primer sets, the 16S rRNA gene was amplified and sequenced.

Of the 32 presumptive *E. faecalis* VRE that were recovered from freezer stock, 25 were confirmed to be *E. faecalis* by *ddl* PCR, and seven were not confirmed by *E. faecalis ddl* PCR. Of these seven latter strains, six were found to be *E. faecium* by *ddl* PCR, and one was found to be a presumptive *Enterococcus raffinosus* or *Enterococcus gilvus* by 16S rRNA gene sequence analysis (99.78 % similarity). In total, 78 % of the colonies predicted to be *E. faecalis* based on Spectra VRE colony colour were confirmed to be *E. faecalis* using *ddl* typing (Table S2).

Of the 55 presumptive *E. faecium* VRE that were recovered from freezer stock, 47 were confirmed to be *E. faecium* by *ddl* PCR, one was a mixed culture of *E. faecium* and *E. faecalis *, and seven were not confirmed by *E. faecium ddl* PCR. All seven of the last group were found to be *E. faecalis* by *ddl* PCR. In total, 85 % of the colonies predicted to be *E. faecium* based on Spectra VRE colony colour were confirmed to be *E. faecium* using *ddl* typing (Table S2).

Of the three atypical white colonies, two were found to be *E. faecium* by *ddl* PCR, and one was found to be *Staphylococcus epidermidis* by 16S rRNA gene sequence analysis (99.85 % similarity) (Table S2).

In summary, of the 90 presumptive VRE isolates analysed using *ddl* typing, 55 were found to be *E. faecium *, 32 were found to be *E. faecalis *, two were found to be neither and one was a mixed culture.

### Determination of vancomycin resistance type and re-testing for growth on Spectra VRE

Vancomycin resistance in enterococci is conferred by the synthesis of peptidoglycan precursors for which vancomycin has reduced binding affinity [13]. Vancomycin resistance loci can be classified by the gene sequences for the d-alanine–d-lactate ligases, VanA and VanB, which are the most prevalent vancomycin resistance types for hospital-associated VRE [14]. We screened our isolates for the presence of *vanA* and *vanB* using previously reported primer sets [3, 4].

Of the 55 *ddl*-confirmed *E. faecium *, 52 were positive for *vanA* and negative for *vanB*, and three were negative for both *vanA* and *vanB* (Table S3). Broth microdilution with vancomycin was performed for 14 of the 52 *vanA*-positive isolates (selected consecutively by patient number) to confirm their vancomycin resistance, and their vancomycin MICs were all ≥256 µg ml^−1^, as expected. The three isolates that were negative for both *vanA* and *vanB* were assessed for growth on Spectra VRE agar, and they did not grow, indicating that the initial clinical cultures were false positives. Broth microdilution with vancomycin was performed for these three isolates. Two of the isolates had vancomycin MICs of 2 µg ml^−1^. One isolate (163-2) had a vancomycin MIC of 512 µg ml^−1^, despite being negative by PCR for both *vanA* and *vanB* and failing to be re-cultured on Spectra VRE agar. Further investigation is required to determine the genetic basis for the phenotypes observed for this strain.

Of the 32 *ddl*-confirmed *E. faecalis *, four were positive for *vanA* and negative for *vanB*, one was positive for *vanB* and negative for *vanA*, and the other 27 were negative for both *vanA* and *vanB* (Table S4). All *ddl*-confirmed *E. faecalis* were assessed for growth on Spectra VRE agar. Only the five *vanA*- or *vanB*-positive strains grew robustly on Spectra VRE agar. For the other 27 strains, no growth was observed, except that a single light blue colony was observed for each of isolates 107-2 and 110. Nine of these 27 *vanA*- and *vanB*-negative strains were also assessed for growth on BHI agar supplemented with vancomycin (6 µg ml^−1^), and they failed to grow. Overall, these results suggest that the initial clinical cultures for 27 of the 32 *E. faecalis* strains were false positives on Spectra VRE agar. To follow up on these results, broth microdilution with vancomycin was performed for five of these isolates (randomly selected from the 27), and their vancomycin MICs were 2–4 µg ml^−1^. Additional investigation is required to determine the mechanism for incongruence between initial clinical culture on Spectra VRE agar and subsequent molecular typing and Spectra VRE re-culture results.

Finally, the presumptive *E. raffinosus/E. gilvus* isolate was found to be *vanA*-positive and *vanB*-negative (Table S5). This isolate was assessed for growth on Spectra VRE agar, and it grew, forming light purple colonies. The broth microdilution vancomycin MIC for this strain was 128 µg ml^−1^.

### MLST and CRISPR2 analysis of select *E. faecalis* isolates

We used multi-locus sequence typing (MLST) [9] and a previously published CRISPR-based typing method for *E. faecalis* [12] to assess phylogenetic relationships among a subset of the *E. faecalis* isolates. MLST uses sequence variation in seven housekeeping loci to characterize phylogenetic relationships between strains. CRISPRs are hypervariable loci consisting of short (36 bp) repeats interspersed by short (30 bp) spacer sequences in *E. faecalis* [10, 11]. Comparative analysis of CRISPR spacer sequences is an alternative method to MLST to analyse relationships between strains [15].

The ST of 11 *E. faecalis* isolates was determined, including two *vanA*-positive isolates and the single *vanB*-positive isolate. Eight STs were identified (Table 1). Five of the 11 isolates were novel STs (ST777, ST778, ST779), including the two *vanA* isolates (both ST779) and the *vanB* isolate (ST778). ST16 (two isolates) and ST179 (one isolate) are single-locus variants of each other. Otherwise, all strains varied at three or more of the seven loci relative to each other.

**Table 1.** MLST and CRISPR typing results

Isolate ID	vanA/B*	ST†	CRISPR2‡	CRISPR-Cas§
2-1	–	59	91-110-108-109-107	Not present
43-2	–	64	85-83-86-82	Not present
59	*vanA*	779	92	Not present
80	–	nd	85-83-86-82	Not present
81	–	nd	12–67	Not present
94-1	–	179	85-83-86-82	Not present
101-2	–	6	12–67	Not present
106	–	nd	85-83-86-82	Not present
107-2	–	nd	1-5-38-70	CRISPR1-Cas
110	–	nd	12-171(SNP)−170-89-169	Not present
119-1	–	16	12-29-28-27-26-25-24-23-22-21	CRISPR1-Cas
122-2	*vanA*	nd	12–67	Not present
125-1	–	nd	85-83-86-82	Not present
127-1	–	nd	85-83-86-82	Not present
131-1	*vanA*	779	92	Not present
132	–	nd	1-5-4-3-2	Not present
133-1	–	777	85-a-b-c-d-e-f-g-h	Not present
141-2	*vanB*	778	1-5-4-3-2	Not present
142-1	–	777	85-i-b-j-e-f-g-h	Not present
142-2	–	16	12-29-28-27-26-25-24-23-22-21	Not present
143-1	–	nd	85-83-86-82	Not present
153-1	–	nd	92	Not present
152	–	nd	85-83-86-82	Not present

*–, negative results for *vanA* and *vanB* PCRs.

†nd, not determined.

‡Numbering scheme is from Hullahalli *et al.* [12]. Letters indicate novel CRISPR spacers. SNP, differs from spacer sequence 171 by a single nucleotide polymorphism.

§The presence or absence of CRISPR1-Cas and CRISPR3-Cas was evaluated for all isolates shown.

The CRISPR2 arrays of 23 *E. faecalis* isolates were amplified, sequenced and analysed (Table 1). We compared the CRISPR2 spacer sequences obtained here with our previous analysis of CRISPR2 loci in 228 *E. faecalis* genomes, wherein spacers of unique sequence were assigned unique numerical identifiers [12]. We identified 12 CRISPR2 types among the 23 isolates. Only two of the 23 isolates analysed here possessed novel spacer sequences relative to our previous study (denoted as letters in Table 1). Eight of the 23 isolates, corresponding to eight patients, had identical CRISPR2 loci of spacers 85-83-86-82.

We also determined the occurrence of the previously identified CRISPR-Cas loci, CRISPR1-Cas and CRISPR3-Cas [10, 11], among the 23 *E. faecalis* analysed for CRISPR2 typing (Table 1). Both of these systems can protect *E. faecalis* from the acquisition of mobile genetic elements that confer antibiotic resistance [16, 17]. No isolates possessed CRISPR3-Cas. Two of the 23 isolates possessed CRISPR1-Cas (Table 1).

Two *E. faecalis* isolates from patient number 142 were analysed by both MLST and CRISPR2 typing (Table 1). The isolates were of different STs and different CRISPR2 types, demonstrating co-colonization of the patient 142 gastrointestinal tract by genetically distinct *E. faecalis *.

## Conclusions

We report the collection and initial analysis of presumptive VRE obtained from hospitalized patients in Dallas, Texas, USA. We determined that most (52 of 55; 94.5 %) of *ddl*-confirmed *E. faecium* from Spectra VRE rectal swab cultures were *vanA*-positive, while most (27 of 32; 84 %) of *ddl*-confirmed *E. faecalis* were negative for both *vanA* and *vanB*. All of the *vanA*- and *vanB*-negative strains failed to be recultured on Spectra VRE agar, with the exception of a single colony observed for each of two *E. faecalis* strains. Conversely, control VRE strains and six *vanA*- and *vanB*-positive rectal swab strains were robustly cultured on Spectra VRE agar, as expected. There were key differences between use of the Spectra VRE agar in the clinical microbiology versus research labs. First, different batches of the agar were used. Second, the inoculation methods differed. In the clinical microbiology lab, rectal swabs (mixed cultures) were used to inoculate the agar, while in the research lab, high-density pure cultures were spread on the agar. That said, of the 30 total isolates that failed to be recultured on Spectra VRE agar, we determined vancomycin MIC for eight of them, and seven had vancomycin MICs of 2–4 µg ml^−1^. The concentration of vancomycin in Spectra VRE is 6 µg ml^−1^, and therefore it is expected that these strains should not grow on this medium. A final point is that in the clinical microbiology lab, the results of the entire agar culture are used to guide surveillance decisions, while in our research study, we selected only one to three representative colonies of different colour and/or morphologies per plate for analysis. Therefore, we cannot comment on whether the total Spectra VRE rectal swab culture comprised vancomycin-sensitive strains, or merely certain colonies, nor can we comment on the overall sensitivity and specificity of Spectra VRE agar for assessing true VRE colonization of these patients. That said, we are not the first to observe false positives using Spectra VRE agar, particularly for *E. faecalis *. In a previous study, patient age was significantly correlated with false positive vancomycin-resistant *E. faecalis* results [18].

The limitations of our study are that we relied primarily on molecular, PCR-based techniques (*ddl* typing; *vanB* and *vanB* amplification) to analyse presumptive VRE, with phenotypic testing on only a subset of isolates, specifically vancomycin MIC testing by broth microdilution. Genome sequencing and more phenotypic assessments of vancomycin susceptibility and heterogeneity will be used in future studies to characterize these isolates further, specifically to potentially identify a mechanistic basis for the presumptive false positives observed. More broadly, follow-on molecular and phenotypic studies of surveillance cultures such as these are clinically relevant. Patients could be falsely labelled as colonized with VRE and require additional isolation precautions which would increase use of personal protective equipment when not needed (this is also associated with increased healthcare costs). There could also be an impact on empiric antibiotic selection, where if infection is suspected, the antibiotic chosen would need to cover VRE and would be inappropriately guided by the false positive results.

In summary, we present a collection of faecal enterococcal isolates from the Dallas area. Most VRE identified via Spectra VRE agar were confirmed to be VanA-type *E. faecium *.

## Supplementary Data

Supplementary material 1
