# Supplementary material for: Characterization of presumptive vancomycin-resistant enterococci recovered during infection control surveillance in Dallas, Texas, USA
Source: Access Microbiol. 2021 Mar 22;3(3):000214. doi: 10.1099/acmi.0.000214 (PMC8209702; doi:10.1099/acmi.0.000214)
Supplement: Supplementary material 1 [file acmi-3-0214-s001.pdf]

Table S1. Primers used in this study. See materials and methods section for references for primers.

Primer name	Sequence (5'-->3')[a]
**Vancomycin resistance typing**	
vanA F	GGGAAAACGACAATTGC
vanA R	GTACAATGCGGCCGTTA
vanB F	ATGGGAAGCCGATAGTC
vanB R	GATTTCGTTCCTCGACC
**Species determination**	
E. faecium ddl F	TAGAGACATTGAATATGCC
E. faecium ddl R	CATCGTGTAAGCTAACTTC
E. faecalis ddl F	ATCAAGTACAGTTAGTCTT
E. faecalis ddl R	ACGATTCAAAGCTAACTG
16S rRNA 8F	AGAGTTTGATCCTGGCTCAG
16S rRNA 1492R	GGTTACCTTGTTACGACTT
**CRISPR-Cas analysis**	
CRISPR2 seq F	CTGGCTCGCTGTTACAGCT
CRISPR2 seq R	GCCAATGTTACAATATCAAACA
CRISPR1-Cas flank F	GCGATGTTAGCTGATACAAC
CRISPR1-Cas flank R	CGAATATGCCTGTGGTGAAA
CRISPR1-Cas *cas9* F	CAGAAGACTATCAGTTGGTG
CRISPR1-Cas *cas9* R	CCTTCTAAATCTTCTTCATAG
CRISPR3-Cas flank F	GATCACTAGGTTCAGTTATTTC
CRISPR3-Cas flank R	CATCGATTCATTATTCCTCCAA
CRISPR3-Cas *cas9* F	GCTGAATCTGTGAAGTTACTC
CRISPR3-Cas *cas9* R	CTGTTTTGTTCACCGTTGGAT
***E. faecalis* MLST**	
gdh-1	GGCGCACTAAAAGATATGGT
gdh-2	CCAAGATTGGGCAACTTCGTCCCA
gyd-1	CAAACTGCTTAGCTCCAATGGC
gyd-2	CATTTCGTTGTCATACCAAGC
pstS-1	CGGAACAGGACTTTCGC
pstS-2	ATTTACATCACGTTCTACTTGC
gki-1	GATTTTGTGGGAATTGGTATGG
gki-2	ACCATTAAAGCAAAATGATCGC
aroE-1	TGGAAAACTTTACGGAGACAGC
aroE-2	GTCCTGTCCATTGTTCAAAAGC
xpt-1	AAAATGATGGCCGTGTATTAGG
xpt-2	AACGTCACCGTTCCTTCACTTA
yiqL-1	CAGCTTAAGTCAAGTAAGTGCCG
yiqL-2	GAATATCCCTTCTGCTTGTGCT

Table S2. *ddl* typing data for presumptive VRE obtained from Spectra VRE plates.

Notes:
* For PCR analysis, an empty cell indicates a negative result for the PCR reaction.
NT indicates Not Tested. n/a indicates that the PCR reaction was not performed because the strain could not be recovered from freezer stock.

					**PCR analysis***				
Patient ID #	Isolate ID #	Colony Color on Spectra VRE	Predicted species based on colony color	Correct prediction?	Efm-*ddl*	Efs-*ddl*	*vanA*	*vanB*	Isolate ID #
1	1	navy blue	*E. faecium*	yes	+		+		1
2	2−1	light blue	*E. faecalis*	yes		+			2−1
	2−2	light blue	*E. faecalis*	yes		+			2−2
	2−3	navy blue	*E. faecium*	no		+			2−3
5	5	navy blue	*E. faecium*	yes	+		+		5
7	7	purple	*E. faecium*	yes	+		+		7
9	9−1	purple	*E. faecium*	yes	+		+		9−1
	9−2	navy blue	*E. faecium*	yes	+		+		9−2
16	16−1	navy blue	*E. faecium*	yes	+		+		16−1
	16−2	light blue	*E. faecalis*	no	+		+		16−2
17	17−1	navy blue	*E. faecium*	yes	+		+		17−1
	17−2	white	---	no	+		+		17−2
20	20	navy blue	*E. faecium*	yes	+		+		20
42	42	navy blue	*E. faecium*	yes	+		+		42
43	43−1	navy blue	*E. faecium*	yes	+		+		43−1
	43−2	light blue	*E. faecalis*	yes		+			43−2
51	51−1	navy blue	*E. faecium*	yes	+		+		51−1
	51−3	light blue	*E. faecalis*	no	+		+		51−3
	51−4	purple	*E. faecium*	yes	+		+		51−4
52	52	navy blue	*E. faecium*	yes	+		+		52
53	53−1	purple	*E. faecium*	yes	+		+		53−1
	53−2	purple (small color	*E. faecium*	yes	+		+		53−2
55	55	navy blue	*E. faecium*	yes	+		+		55
57	57	navy blue	*E. faecium*	yes	+		+		57
59	59	light blue	*E. faecalis*	yes		+	+		59
60	60	navy blue	*E. faecium*	mixed culture	+	+	+	+	60
66	66	navy blue	*E. faecium*	yes	+		+		66
70	70	purple	*E. faecium*	yes	+				70
71	71−1	navy blue	*E. faecium*	yes	+		+		71−1
	71−2	purple	*E. faecium*	yes	+		+		71−2
76	76	navy blue	*E. faecium*	not revived from stock	n/a	n/a	n/a	n/a	76
80	80	light blue	*E. faecalis*	yes		+			80
81	81	light blue	*E. faecalis*	yes		+			81
83	83	navy blue	*E. faecium*	yes	+		+		83
87	87−1	navy blue	*E. faecium*	yes	+		+		87−1
	87−2	purple	*E. faecium*	not revived from stock	n/a	n/a	n/a	n/a	87−2
88	88	navy blue	*E. faecium*	not revived from stock	n/a	n/a	n/a	n/a	88
91	91	navy blue	*E. faecium*	yes	+		+		91
93	93−1	navy blue	*E. faecium*	yes	+		+		93−1
	93−2	white	---	no	+		+		93−2
	93−3	light blue	*E. faecalis*	no	+		+		93−3
94	94-1	light blue	*E. faecalis*	yes		+			94-1
	94-2	big light blue	*E. faecalis*	yes		+			94-2
97	97−1	purple	*E. faecium*	yes	+		+		97−1
	97−2	navy blue	*E. faecium*	yes	+		+		97−2

100	100-1	navy blue	*E. faecium*	yes	+		+			100-1
	100-2	light blue	*E. faecalis*	no	+		+			100-2
101	101-1	large light blue	*E. faecalis*	yes		+				101-1
	101-2	light blue	*E. faecalis*	yes		+				101-2
103	103	navy blue	*E. faecium*	yes	+		+			103
106	106	light blue	*E. faecalis*	yes		+				106
107	107-1	navy blue	*E. faecium*	yes	+		+			107-1
	107-2	light blue	*E. faecalis*	yes		+				107-2
110	110	light blue	*E. faecalis*	yes		+				110
111	111	navy blue	*E. faecium*	yes	+		+			111
113	113	navy blue	*E. faecium*	yes	+		+			113
119	119−1	navy blue	*E. faecium*	no		+				119−1
	119−2	dark blue smudge	*E. faecium*	no		+				119−2
121	121	navy blue	*E. faecium*	yes	+		+			121
122	122-1	navy blue	*E. faecium*	no		+	+			122-1
	122-2	light blue	*E. faecalis*	yes		+	+			122-2
124	124-1	navy blue	*E. faecium*	yes	+		+			124-1
	124-2	purple	*E. faecium*	yes	+		+			124-2
	124-3	light blue	*E. faecalis*	no	+		+			124-3
125	125-1	light blue	*E. faecalis*	yes		+				125-1
	125-2	small light blue	*E. faecalis*	yes		+				125-2
127	127-1	light blue	*E. faecalis*	yes		+				127-1
	127-2	large light blue	*E. faecalis*	yes		+				127-2
131	131-1	light blue	*E. faecalis*	yes		+	+			131-1
	131-2	purple	*E. faecium*	yes	+		+			131-2
132	132	navy blue	*E. faecium*	no		+				132
133	133-1	navy blue	*E. faecium*	no		+				133-1
	133-2	white	---	no - *Staphylococcus epidermidis* by 16S rRNA			NT	NT		133-2
136	136-1	navy blue	*E. faecium*	yes	+		+			136-1
137	137	navy blue	*E. faecium*	yes	+		+			137
141	141-1	light blue	*E. faecalis*	not revived from stock	n/a	n/a	n/a	n/a		141-1
	141-2	navy blue	*E. faecium*	no		+		+		141-2
142	142-1	light blue	*E. faecalis*	yes		+				142-1
	142-2	light blue (small)	*E. faecalis*	yes		+				142-2
143	143-1	light blue	*E. faecalis*	yes		+				143-1
	143-2	small light blue	*E. faecalis*	yes		+				143-2
144	144-1	navy blue	*E. faecium*	yes	+		+			144-1
	144-2	purple	*E. faecium*	not revived from stock	n/a	n/a	n/a	n/a		144-2
145B	145B	navy blue	*E. faecium*	not revived from stock	n/a	n/a	n/a	n/a		145B
146	146	light blue	*E. faecalis*	no - *E. raffinosus* or *E. gilvus* by 16S rRNA			+			146
148	148	navy blue	*E. faecium*	yes	+		+			148
151	151-1	purple	*E. faecium*	not revived from stock	n/a	n/a	n/a	n/a		151-1
	151-2	navy blue	*E. faecium*	not revived from stock	n/a	n/a	n/a	n/a		151-2
152	152	light blue	*E. faecalis*	yes		+				152
153	153-1	light blue	*E. faecalis*	yes		+				153-1
	153-2	purple	*E. faecium*	not revived from stock	n/a	n/a	n/a	n/a		153-2
154	154-1	navy blue	*E. faecium*	yes	+		+			154-1
	154-2	purple	*E. faecium*	not revived from stock	n/a	n/a	n/a	n/a		154-2
155	155	navy blue	*E. faecium*	yes	+		+			155
158	158	navy blue	*E. faecium*	yes	+		+			158
160	160	navy blue	*E. faecium*	yes	+		+			160
161	161	navy blue	*E. faecium*	yes	+		+			161
162	162	navy blue	*E. faecium*	yes	+		+			162
163	163-1	navy blue	*E. faecium*	yes	+					163-1
	163-2	light blue	*E. faecalis*	no	+					163-2

Table S3. Data for *ddl*-confirmed *E. faecium* strains.

Notes:
* For PCR analysis, an empty cell indicates a negative result for the PCR reaction.
NT indicates Not Tested. n/a indicates that the PCR reaction was not performed because the strain could not be recovered from freezer stock.
** For Spectra VRE re-testing, a blank cell indicates that the experiment was not performed for that strain.
*** Two trials of broth microdilution were performed. If different MIC values were obtained across the two trials, both values are stated.
A blank cell indicates that the experiment was not performed for that strain.

					PCR analysis*						
Patient ID #	Isolate ID #	Colony Color on Spectra VRE	Predicted species based on colony color	Correct prediction?	Efm-*ddl*	Efs-*ddl*	*vanA*	*vanB*	Re-test for growth on Spectra VRE agar**	Vancomycin MIC by broth microdilution***	Isolate ID #
1	1	navy blue	*E. faecium*	yes	+		+			1024	1
5	5	navy blue	*E. faecium*	yes	+		+			2048	5
7	7	purple	*E. faecium*	yes	+		+			512	7
9	9−1	purple	*E. faecium*	yes	+		+			2048	9−1
	9−2	navy blue	*E. faecium*	yes	+		+				9−2
16	16−1	navy blue	*E. faecium*	yes	+		+			1024	16−1
	16−2	light blue	*E. faecalis*	no	+		+				16−2
17	17−1	navy blue	*E. faecium*	yes	+		+			512/1024	17−1
	17−2	white	---	no	+		+				17−2
20	20	navy blue	*E. faecium*	yes	+		+			1024	20
42	42	navy blue	*E. faecium*	yes	+		+			1024	42
43	43−1	navy blue	*E. faecium*	yes	+		+			2048	43−1
51	51−1	navy blue	*E. faecium*	yes	+		+			2048	51−1
	51−3	light blue	*E. faecalis*	no	+		+			1024	51−3
	51−4	purple	*E. faecium*	yes	+		+			1024	51−4
52	52	navy blue	*E. faecium*	yes	+		+			2048	52
53	53−1	purple	*E. faecium*	yes	+		+			256	53−1
	53−2	purple (small color	*E. faecium*	yes	+		+				53−2
55	55	navy blue	*E. faecium*	yes	+		+				55
57	57	navy blue	*E. faecium*	yes	+		+				57
66	66	navy blue	*E. faecium*	yes	+		+				66
70	70	purple	*E. faecium*	yes	+				No growth	2	70
71	71−1	navy blue	*E. faecium*	yes	+		+				71−1
	71−2	purple	*E. faecium*	yes	+		+				71−2
83	83	navy blue	*E. faecium*	yes	+		+				83
87	87−1	navy blue	*E. faecium*	yes	+		+				87−1
91	91	navy blue	*E. faecium*	yes	+		+				91
93	93−1	navy blue	*E. faecium*	yes	+		+				93−1
	93−2	white	---	no	+		+				93−2
	93−3	light blue	*E. faecalis*	no	+		+				93−3
97	97−1	purple	*E. faecium*	yes	+		+				97−1
	97−2	navy blue	*E. faecium*	yes	+		+				97−2
100	100-1	navy blue	*E. faecium*	yes	+		+				100-1
	100-2	light blue	*E. faecalis*	no	+		+				100-2
103	103	navy blue	*E. faecium*	yes	+		+				103
107	107-1	navy blue	*E. faecium*	yes	+		+				107-1
111	111	navy blue	*E. faecium*	yes	+		+				111
113	113	navy blue	*E. faecium*	yes	+		+				113
121	121	navy blue	*E. faecium*	yes	+		+				121
124	124-1	navy blue	*E. faecium*	yes	+		+				124-1
	124-2	purple	*E. faecium*	yes	+		+				124-2
	124-3	light blue	*E. faecalis*	no	+		+				124-3
	131-2	purple	*E. faecium*	yes	+		+				131-2
136	136-1	navy blue	*E. faecium*	yes	+		+				136-1
137	137	navy blue	*E. faecium*	yes	+		+				137
144	144-1	navy blue	*E. faecium*	yes	+		+				144-1
148	148	navy blue	*E. faecium*	yes	+		+				148
154	154-1	navy blue	*E. faecium*	yes	+		+				154-1
155	155	navy blue	*E. faecium*	yes	+		+				155
158	158	navy blue	*E. faecium*	yes	+		+				158
160	160	navy blue	*E. faecium*	yes	+		+				160
161	161	navy blue	*E. faecium*	yes	+		+				161
162	162	navy blue	*E. faecium*	yes	+		+				162
163	163-1	navy blue	*E. faecium*	yes	+				No growth	2	163-1
	163-2	light blue	*E. faecalis*	no	+				No growth	512	163-2

Table S4. Data for *ddl*-confirmed *E. faecalis* strains.

Notes:
* For PCR analysis, an empty cell indicates a negative result for the PCR reaction.
NT indicates Not Tested. n/a indicates that the PCR reaction was not performed because the strain could not be recovered from freezer stock.
** For Spectra VRE re-testing, a blank cell indicates that the experiment was not performed for that strain.
*** Two trials of broth microdilution were performed. A blank cell indicates that the experiment was not performed for that strain.

					**PCR analysis***							
Patient ID #	Isolate ID #	Colony Color on Spectra VRE	Predicted species based on colony color	Correct prediction?	Efm-*ddl*	Efs-*ddl*	*vanA*	*vanB*	Re-test for growth on Spectra VRE agar**	Growth on BHI + 6 ug/ml Van	Vancomycin MIC by broth microdilution***	Isolate ID #
2	2−1	light blue	*E. faecalis*	yes		+			No growth			2−1
	2−2	light blue	*E. faecalis*	yes		+			No growth	No growth	4 ug/ml	2−2
	2−3	navy blue	*E. faecium*	no		+			No growth	No growth		2−3
	43−2	light blue	*E. faecalis*	yes		+			No growth			43−2
59	59	light blue	*E. faecalis*	yes		+	+		Growth - light blue colonies		>= 512 ug/ml	59
80	80	light blue	*E. faecalis*	yes		+			No growth			80
81	81	light blue	*E. faecalis*	yes		+			No growth			81
94	94-1	light blue	*E. faecalis*	yes		+			No growth			94-1
	94-2	big light blue	*E. faecalis*	yes		+			No growth	No growth		94-2
101	101-1	large light blue	*E. faecalis*	yes		+			No growth			101-1
	101-2	light blue	*E. faecalis*	yes		+			No growth	No growth		101-2
106	106	light blue	*E. faecalis*	yes		+			No growth			106
	107-2	light blue	*E. faecalis*	yes		+			1 light blue colony		4 ug/ml	107-2
110	110	light blue	*E. faecalis*	yes		+			1 light blue colony		2 ug/ml	110
119	119−1	navy blue	*E. faecium*	no		+			No growth			119−1
	119−2	dark blue smudge	*E. faecium*	no		+			No growth	No growth		119−2
122	122-1	navy blue	*E. faecium*	no		+	+		Growth - navy blue colonies	Growth		122-1
	122-2	light blue	*E. faecalis*	yes		+	+		Growth - light blue colonies			122-2
125	125-1	light blue	*E. faecalis*	yes		+			No growth			125-1
	125-2	small light blue	*E. faecalis*	yes		+			No growth	No growth	2 ug/ml	125-2
127	127-1	light blue	*E. faecalis*	yes		+			No growth			127-1
	127-2	large light blue	*E. faecalis*	yes		+			No growth	No growth		127-2
131	131-1	light blue	*E. faecalis*	yes		+	+		Growth - light blue colonies			131-1
132	132	navy blue	*E. faecium*	no		+			No growth		2 ug/ml	132
133	133-1	navy blue	*E. faecium*	no		+			No growth			133-1
	141-2	navy blue	*E. faecium*	no		+		+	Growth - light blue colonies			141-2
142	142-1	light blue	*E. faecalis*	yes		+			No growth			142-1
	142-2	light blue (small)	*E. faecalis*	yes		+			No growth	No growth		142-2
143	143-1	light blue	*E. faecalis*	yes		+			No growth			143-1
	143-2	small light blue	*E. faecalis*	yes		+			No growth	No growth		143-2
152	152	light blue	*E. faecalis*	yes		+			No growth			152
153	153-1	light blue	*E. faecalis*	yes		+			No growth			153-1

Table S5. Data for species recovered other than *E. faecalis* or *E. faecium* .

Notes:
* For PCR analysis, an empty cell indicates a negative result for the PCR reaction.
NT indicates Not Tested. n/a indicates that the PCR reaction was not performed because the strain could not be recovered from freezer stock.
** For Spectra VRE re-testing, a blank cell indicates that the experiment was not performed for that strain.
*** Two trials of broth microdilution were performed. A blank cell indicates that the experiment was not performed for that strain.

					PCR analysis*							
Patient ID #	Isolate ID #	Colony Color on Spectra VRE	Predicted species based on colony color	Correct prediction?	Efm-*ddl*	Efs-*ddl*	*vanA*	*vanB*	Re-test for growth on Spectra VRE agar**	Growth on BHI + 6 ug/ml Van	Vancomycin MIC by broth microdilution***	Isolate ID #
	133-2	white	---	no - *Staphylococcus epidermidis* by 16S rRNA			NT	NT	No growth	No growth		133-2
146	146	light blue	*E. faecalis*	no - *E. raffinosus* or *E. gilvus* by 16S rRNA			+		Growth - light purple colonies		128 ug/ml	146
